# Drug-Induced Photosensitivity: Clinical Types of Phototoxicity and Photoallergy and Pathogenetic Mechanisms

**DOI:** 10.3389/falgy.2022.876695

**Published:** 2022-06-20

**Authors:** Luca Di Bartolomeo, Natasha Irrera, Giuseppe Maurizio Campo, Francesco Borgia, Alfonso Motolese, Federico Vaccaro, Francesco Squadrito, Domenica Altavilla, Alessandra Grazia Condorelli, Alberico Motolese, Mario Vaccaro

**Affiliations:** ^1^Department of Clinical and Experimental Medicine, Dermatology, University of Messina, Messina, Italy; ^2^Department of Clinical and Experimental Medicine, Pharmacology, University of Messina, Messina, Italy; ^3^Laboratory of Clinical Biochemistry, Department of Clinical and Experimental Medicine, University of Messina, Messina, Italy; ^4^Department of Dermatology, University of Modena and Reggio Emilia, Modena, Italy; ^5^S.C. Dermatologia, Azienda USL di Reggio Emilia-IRCCS, Arcispedale Santa Maria Nuova, Reggio Emilia, Italy

**Keywords:** photosensitivity, phototoxicity, photoallergy, drug reaction, pathogenetic mechanisms, patient education

## Abstract

Drug-induced photosensitivity (DIP) is a common cutaneous adverse drug reaction, resulting from the interaction of ultraviolet radiations, mostly ultraviolet A, with drugs. DIP includes phototoxicity and photoallergy. A phototoxic reaction is obtained when topical and systemic drugs or their metabolites absorb light inducing a direct cellular damage, while a photoallergic reaction takes place when the interaction between drugs and ultraviolet radiations causes an immune cutaneous response. Clinically, phototoxicity is immediate and appears as an exaggerated sunburn, whereas photoallergy is a delayed eczematous reaction. DIP may show several clinical subtypes. In this mini-review we report the pathogenetic mechanisms and causative drugs of DIP. We offer a detailed description of DIP clinical features in its classical and unusual subtypes, such as hyperpigmentation/dyschromia, pseudoporphyria, photo-onycolysis, eruptive teleangiectasia, pellagra-like reaction, lichenoid reaction, photodistributed erythema multiforme and subacute/chronic cutaneous lupus erythematosus. We described how physicians may early recognize and manage DIP, including diagnostic tests to rule out similar conditions. We made suggestions on how to improve sun exposure behaviors of patients at risk of DIP by means of an aware use of sunscreens, protective clothing and recent technologic tools. We highlighted the lack of sun safety programs addressed to patients at risk of DIP, who need a formal education about their condition.

## Introduction

Drug-induced photosensitivity (DIP) is a common cutaneous adverse drug reaction, resulting from the interaction of ultraviolet radiations (UVR) with drugs (1). DIP may account for up to 8% of all cutaneous adverse drug reactions (2). Photosensitive reactions occur mainly in the UVA range (wavelength 315–400 nm), although some drugs produce photosensitivity upon exposure to UVB radiations (280–315 nm) or even visible light (400–740 nm) (3). DIP may be induced by sunlight and artificial sources of UV radiation, such as medical phototherapy lamps, tanning beds, LEDs, UV lasers, light emitting diodes (LEDs) and other lamps used in industry (4). Given the UVA-dependency, DIP may be induced also through window panes at any time of the year. In fact, standard window glass filters UVB, but not UVA (5). The drug or its metabolites' ability to absorb UVR or visible radiation is critical to induce biochemical changes in the tissue. The process is termed “photosensitization” and the initiator is the “photosensitizer” (1). DIP may occur because of systemic or local drugs.

Two types of DIP reactions are distinguished: drug-induced phototoxic reactions (DI-PTRs) and drug-induced photoallergic reactions (DI-PARs). The first are the result of a direct cellular damage, while the second are caused by an immune-mediated mechanism of action (6). Pathogenetic initial stages of DI-PRTs and DI-PARs are similar. Absorption of photons by photosensitizing drug molecules lead them to a more instable reactive excited state, called triplet state (1, 3, 7). Photosensitizers in the excited triplet state lead to production of free radicals or singlet oxygen, which directly damage cell components (8, 9). In DI-PTRs, the drug absorbs energy from UVA light and releases it into the skin, causing cellular damage, while in DI-PARs, light may cause a structural change in a drug, which binds protein and becomes a photoallergen, causing an immune response mediated by T-cells (10, 11). DI-PTRs are dose-dependent, namely are proportionate to drug and light dose. DI-PARs require previous exposure to the photosensitizing agent and appear as a delayed hypersensitivity reaction (1). The reaction is dose-independent. Clinically, photosensitive reactions involve sun-exposed areas, namely the face, V of the neck and extensor, forearms and hands (6). Clinical manifestations of DI-PTRs may onset from 30 min to 24 h after sun exposure and may be transient or lasting up to 4 days, according to the type of photosensitizer (1). DI-PTRs presentation resembles an exaggerated sunburn, mostly presenting with burning and/or painful erythema, edema and vesiculation; while DI-PARs appear some days after exposure with an eczematous itching dermatitis (1). In addition to these classical types, DI-PTRs and DI-PARs may presented several subtypes.

**Figure 1:**
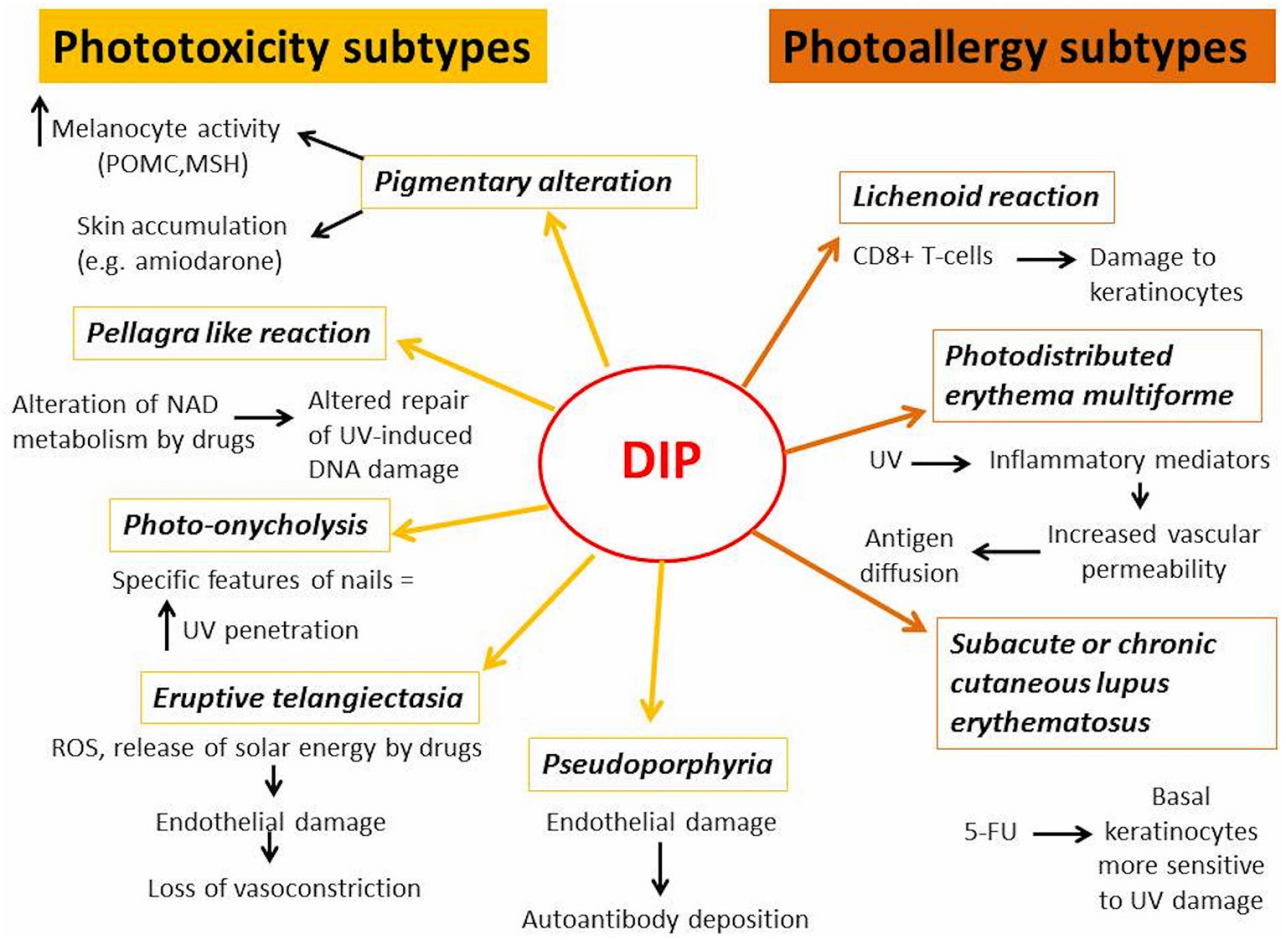
Summary of pathogenetic mechanisms involved in DIP.

In next chapters, the differences between DI-PTRs and DI-PARs' subtypes were described, focusing on their clinical presentation, pathogenetic mechanisms (Figure 1) and causative drugs (Table 1).

**Table 1:** Drugs associated with phototoxicity and photoallergy subtypes.

**Phototoxicity subtypes**	**Associated drugs**
Hyperpigmentation and dyschromia	citalopram (12), imipramine (13), amitriptilyne (14), chlorpromazine (15), diltiazem (16), amiodarone (17–19), vandetanib (20)
Pseudoporphyria	tetracycline (21), nalidixic acid (22), voriconazolo (23, 24), furosemide (25), hydrochlorothiazide/triamterene (26), amiodarone (27), etretinate (28) and isotretinoin (29), olanzapine (30), naprossene (31, 32), imatinib (33)
Photo-onycholysis	doxycycline (34, 35), indapamide (36), voriconazole (24), griseofulvin (37), sparfloxacin (38), olanzapine, aripiprazole (39), benoxyprofen (40), diclofenac (41), photodynamic therapy (42)
Photodistributed telangiectasia	escitalopram (43), cefotaxime (44), venlafaxine (45), mirtazapine and levetiracetam (46), amlodipine (47)
Pellagra like reaction	isoniazid (48), 5-fluorouracil (49), haloperidol (50), azathioprine (51, 52), ethionamide (53)
**Photoallergy subtypes**	**Associated drugs**
Lichenoid reaction	thiazide diuretics (54), quinine (55), quinidine (56), demeclocycline (57), isoniazide (58), sparfloxacin (59), doxycycline (60), tegaful (61), capecitabine (62), chlorpromazine (63), carbamazepine (64), thioridazine (65), naprossene (66), enalapril (67), phenofibrate (68), clopidogrel (69)
Photodistributed erythema multiforme	simvastin and pravastatin (70), paclitaxel (71), naprossene (72), ketoprofene (73), phenylbutazone (74), itraconazolo (75) tocilizumab (76), oxybenzone (77)
Subacute or chronic cutaneous lupus erythematosus	griseofulvin, terbinafine, calcium channel blockers, beta blockers, diuretics (78, 79), proton-pump inhibitors (80), antineoplastic drugs (81), anti-TNFα (82, 83), checkpoint inhibitors (84)

## Phototoxicity Subtypes

### Hyperpigmentation and Dyschromia

Hyperpigmentation of the skin is a common side effect of many drugs (85). It may occur after acute phototoxic reaction or be the sole feature (86). In addition to hyperpigmentation, drug-induced dyschromia may also occur. Discoloration may range from blue-brown to slate-gray. Classical examples of drugs inducing photopigmentation are listed in Table 1 (12–20). Photosensitizing drugs may cause skin hyperpigmentation increasing melanocyte activity and accumulation of melanin or by their accumulation in the skin. In the first case, the drugs could act by amplifying the energy of UV rays and releasing it in well-defined areas of the skin, causing alteration in the production of melanin. Exposure to UVB and inflammatory response may increase melanin production, by regulating cutaneous levels of pro-opiomelanocortin (POMC) mRNA, POMC peptides, and melanocyte stimulating hormone (MSH) receptors (87). On the other hand, chronic accumulation of photosensitive drugs or their metabolites in dermis may determine hyperpigmentation or dyschromia, as in case of amiodarone-induced photosensitivity (18).

Drug-induced photopigmentation should be distinguished from some forms of drug-induced hyperpigmentation that are not only related to solar exposure, such as tetracycline-induced hyperpigmentation. Phototoxicity of tetracyclines is a well-known side effect and it is also part of the mechanism of action, because tetracyclines act as light-activated antibiotics by binding to bacterial cells and killing them upon illumination (88). Tetracycline-induced skin hyperpigmentation may occur as post-inflammatory result of a phototoxic reaction but other mechanisms may explain this adverse event (89).

### Pseudoporphyria

Pseudoporphyria appears as a photodistributed bullous disorder with clinical and histologic features of porphyria cutanea tarda (PCT), without any abnormal porphyrin levels. Pseudoporphyria has been attributed to medications, UVA radiation (tanning beds), excessive sun exposure and chronic renal failure/dialysis (90). Drug-induced pseudoporphyria has been described with many drugs listed in Table 1 (21–32). More recently, imatinib-induced pseudoporphyria has been investigated (33).

Clinically, pseudoporphyria resembles PCT, with vesicles, bullae, skin fragility, milia, and scarring occur on sun-exposed areas. The dorsal hands are most commonly affected, but fingers, extensor legs, upper chest and face may also be involved (90). In contrast to PCT, hypertrichosis, hyperpigmentation, sclerodermoid changes, and dystrophic calcification are rarely reported. Photochemical events of PCT occur after porphyrins absorb light energy in the 400 to 410 nm range (Soret band) while the action spectrum of UVR in pseudoporphyria appears to be in the UVA range (90). Although the pathogenesis of porphyria and pseudoporphyria is still not fully understood, the mechanism involved in the induction of blisters may be similar in the two diseases, involving a physiologic autoantibody reaction to the damaged endothelium, with deposition of IgG and other immunoreactants (91). Nevertheless, the initial skin damage is probably phototoxic and mediated by singlet oxygen formation (92).

### Photo-Onycholysis

Drug-induced photo-onycholysis usually appears at least 2 weeks after initial drug uptake and it may be painful (39, 93). It has been reported with many drugs listed in Table 1 (24, 34–41) and after photodynamic therapy (42).

Four distinct clinical subtypes have been described (93, 94): type I shows a half-moon-shaped separation that is concave distally; type II has a circular notch opened distally and shaped; type III shows a round yellow staining that turns reddish after 5–10 days in the central part of nail bed; in type IV, bullae under the nails have been reported. One common sign is prevalent in the first three types: the lateral margins of the nails are unaffected. Nevertheless, an apparent relationship between responsible drugs and different types of photo-onycholysis is not detectable (93). Photo-onycholysis may be considered a specific sunburn peeling, with elective involvement of nails for two main reasons: the absence of sebaceous glands and of stratum granulosus may enable the penetration of UV because lipids on the skin reduce the UV transmission; the nail's shape may act as a convex lens focusing UV onto the nail bed (39, 93).

### Eruptive Telangiectasia

Iatrogenic telangiectasia is a poorly understood dermatological undesired effect of several drugs (43). Photodistributed telangiectasia has been described with many drugs listed in Table 1 (43–47).

Photopatch tests are usually negative but provocation tests with UVA and UVB may cause the onset of telangiectasia on irradiated skin after 24–48 h, confirming photosensitivity (44).

The damage of endothelial cells in photodistributed telangiectasia may be due to the release of solar energy by photosensitive drug in concentrated way in the skin, that leads to loss of vasoconstrictor function of the vessels; on the other hand, the photodynamic production of ROS would attack directly the endothelial cells compromising their functionality (95). The ability of CCB to generate ROS leading to vasodilatation has been widely investigated. All CCB associated with photodistributed telangiectasia fall into the dihydropyridine group (47).

### Pellagra Like Reaction

Pellagra is caused by a deficiency of niacin and its precursor tryptophan. Clinically, it is characterized by the presence of diarrhea, dementia, and dermatitis (48). The rash usually appears on sun-exposed areas as symmetrical erythema, and, subsequently, as leathery hyperpigmented plaques (48). Pellagra like reactions have been described with drugs listed in Table 1 (48–53). Tryptophan and niacin are precursors of nicotinamide adenine dinucleotide (NAD), a cellular coenzyme involved in repair of UV-induced DNA damage. The drugs leading to pellagrous dermatitis can interfere with niacin/NAD metabolism by inhibiting niacin production from dietary tryptophan and by acting as NAD analogs due to their structural similarity (48).

## Photoallergy Subtypes

### Lichenoid Reaction

Lichenoid reactions appear as scaling violaceous erythema or violaceous papules with Wickham's striae on sun exposed areas, without involvement of mucous membranes (96). Photosensitive drug-induced lichenoid reactions have been described with drugs listed in Table 1 (54–65, 67–69, 72). CD8+ T-cells would be involved in lichenoid reaction, infiltrating the upper dermis and causing inflammatory damage to keratinocytes (58, 59). It is not clear whether lichenoid lesions are the result of a phototoxic or photoallergic reaction, but the long incubation period and the positivity of photopatch test seem to confirm their allergic nature (58, 59).

### Photodistributed Erythema Multiforme

Erythema multiforme (EM) is a muco-cutaneous hypersensitivity reaction frequently triggered by infections or drugs. EM appears as a polymorphous eruption of macules, papules, and characteristic target lesions in symmetrical distribution with a propensity for acral sites (97). The term photodistributed erythema multiforme (PEM) is used to designate a particular form of EM characterized by lesions that are confined to sun-exposed areas, with a clear detachment from unexposed areas. PEM may be triggered more frequently by herpes simplex virus infections and drugs (98). Drug-induced PEM has been described with many drugs listed in Table 1 (70–76). Drug-induced PEM has been reported also with oxybenzone, an UV-absorbing agent used incommercially-available sunscreen (77). Drug-induced PEM appears as a delayed reaction, and even ten days may pass from sun exposure to clinical manifestations (77). In PEM, UV radiation may facilitate the diffusion of skin antigens into the blood stream by inducing the release of inflammatory mediators, such as quinines, prostaglandins and histamine, which increase vascular permeability (98). Either a photoproduct or the actual drug activated by UV radiation might act as an antigen, triggering the immune response, or as a phototoxic agent, favoring the rupture of cells and the release of nuclear antigens (98).

### Subacute or Chronic Cutaneous Lupus Erythematosus

Drug-induced lupus erythematosus (DILE) is defined as an entity characterized by clinical manifestations and immune-pathological serum findings similar to those of idiopathic lupus but which is related to continuous drug exposure and resolves after discontinuation of the offending drug (99). Drug-induced subacute cutaneous lupus erythematous (DI-SCLE) is characterized by annular polycyclic and/or papulosquamous lesions and frequent presence of anti-Ro/SSA antibodies, but the incidence of anti-Ro/SSA antibodies appears to be lower than in idiopathic SCLE. Antihistone antibodies are uncommonly found in DI-SCLE (99).

DI-SCLE has been described with drugs listed in Table 1 (78, 79). Drug-induced discoid lupus erythematous (DI-DLE) is very rarely described in the literature. Clinical appearance is characterized by classic erythematous and scaly discoid lesions, but aspects of lupus tumidus can occasionally develop (99). DI-DLE has been described with drugs listed in Table 1 (81–84).

Several mechanisms are involved in DILE, including molecular mimicry, direct cytotoxicity, disruption of central immune tolerance or altered T-cell function due to hypomethylation and most of them may be light-independent (99). In the cases of cutaneous DILE associated with 5-fluorouracil, it has been suggested that the drug may alter basal keratinocytes, making them more sensitive to ultraviolet damage (99).

## Physicians Education

Physicians should be able to early recognize DIP, distinguishing between DI-PTRs and DI-PARs and to rule out other photosensitive dermatoses that may mimic some unusual clinical subtypes of DIP, such as cutaneous porphyria, pellagra, EM, SCLE, DLE. The diagnosis of DIP is suggested by the photodistributed nature of the eruption and a history of exposure to a topical or systemic photosensitizer. It may be confirmed by monochromator phototesting and photopatch tests (58, 100). Other tests, including laboratory assessment and histological examination for photosensitive dermatosis (e.g., lupus erythematous, porphyria), may be performed to rule out other causes of photodistributed eruptions (78, 99). A large number of medications may cause photosensitivity and in patients using multiple drugs (e.g., elderly) the identification of the responsible drug can be challenging. Treatment is based on the withdrawal of the offending drug and sun protection, especially against UVA wavelengths. Severe symptomatic reactions may need a short course of topical or systemic steroids treatment (44, 100). If a drug is indispensable to the patient, dose reduction and/or sun avoidance with photo-protective measures may avoid discontinuing that drug (101–104).

Moreover, physicians prescribing photosensitizing drugs, such as those described so far, should always inform patients about their possible side effects and advise them appropriate sun protection measures.

## Patients Education

Patients awareness about photosensitizing potential of some drugs is important for primary and secondary prevention of DIP. The improvement of sun exposure behaviors is essential in education of patients taking photosensitizing drugs. The best way to protect these patients from the development of DIP is avoiding sun exposure. When it is not possible, namely in the majority of cases, sun protection education is essential and is based on the use of sunscreens, protective clothing and, more recently, technologic tools such some smartphone apps.

Patients should be educated to use sunscreens filtering not only UVB but also the whole UVA spectrum, because DIPs are mainly caused by light with wavelength 315–400 nm, as mentioned above. Broad-spectrum sunscreens, protecting from both UVB and UVA, are preferred and should have a high sun protection factor, namely 50 or higher. They should be applied before sun exposure and reapplied once within 1 h (101).

Patients should always wear covering clothing, wide-brimmed hats and sunglasses when going outside. Nevertheless, the spaces between the fibers of woven textiles may allow the UV to permeate (102). Patients should take into account this possibility when choosing clothes to wear. The ultraviolet protection factor (UPF) is a measure of ultraviolet radiation penetration through the fabric (103). The UPF of clothing depends on fabric components, including fiber content, color, and additives, and may change with the wear of the fabric over time. Patients at risk of DIP should prefer clothing with an UPF of 40 or higher (103, 104).

Patients exposed to high levels of solar radiation, for occupational or environmental reasons, should be especially careful while taking photosensitizing drugs: that's the case of travelers taking doxycycline for prophylaxis of malaria, which is endemic in tropical countries close to equator (105). The measurement of UV levels is a common function of sun protection smartphone apps and make patients more ready to improve their sun exposure behaviors. Moreover, a multitude of applications also provides tailored recommendations to patients and reminders for protecting their skin, including what type of sunscreen to use, after how long to reapply it, and what kinds of physical protection to dress (i.e., clothing, hats, and sunglasses) (106).

Sun safety education programs are not very common among patients at risk of photosensitivity, although they need it. Huang et al. (107) demonstrated the role of sun protection education in photosensitive patients, specifically suffering from chronic actinic dermatitis or polymorphous light eruption. The patients improved their sun exposure habits and quality of life after intensive educational lessons, combined with an instruction manual. Moreover, the Authors highlighted that routine instruction on sun protection from dermatologists are insufficient for patients at risk of photosensitivity to develop a real awareness of their condition and an appropriate attention to sun safety (107). Considering the efficacy of sun safety educations programs in other many risk categories, such as children (108) or outdoor workers (109), it appears appropriate that similar awareness campaigns will be designed and implemented for patients at risk of DIP.

## Discussion

DIP is an underdiagnosed clinical entity, mainly for two reasons: DIP in atypical presentations is often not recognized and the reaction vanishes within a few days without an explanation; in most cases, especially in patients using multiple drugs, it's difficult to find the culprit drug. A proper knowledge of clinical subtypes of phototoxic and photoallergic drug-induced reactions and of drugs directly involved in these reactions may increase diagnostic accuracy of physicians.

Nevertheless, some issues concerning DIP remain unresolved, such as the long-term effects of DIP. Increasing reports show that continuation of the some phototoxic drug long-term may induce photocarcinogenesis, as in the case of voriconazole (24). Several pathways would be involved in carcinogenesis by photo-oxidation. UVR activates epidermal growth factor receptor (EGFR), a critical mediator of several types of epithelial cancers, and the generation of ROS upregulates the tyrosine kinase activity of the EGFR (110, 111). In addition to EGFR, ROS produced during DIP may activate several signal transduction cascades such as mitogen-activated protein kinases (MAPK), involved in proliferation and antiapoptotic signaling of many cancers, and lead to genomics instability and DNA damage, which have a pro-tumorigenic effect (112, 113). Further studies are needed to better elucidate these mechanisms in DIP. Moreover, it would be interesting to investigate if, as in other skin pathologies involving ROS and immune system (e.g., vitiligo), the pathogenesis of DIP is based on relational networks rather than separate pathways (114–117).

Another issue concerns the education of patients with DIP. Huang et al. demonstrated that sun safety programs may improve sun exposure habits and quality of life of photosensitive patients, but the efficacy of similar campaigns has not yet been evaluated in DIP patients (107). Sun safety programs for DIP patients appear essential, considering that currently an appropriate sun protection education is the only way to prevent DIP and the only alternative therapy to drug withdrawal.

In conclusion, many issues regarding prevention, treatment and prognosis of DIP are unanswered. To date, the control of DIP is based on prompt recognition of its clinical subtypes by dermatologists and sun-exposure education of patients using drugs with potential photosensitivity.

## Author Contributions

MV and FV conceived the idea to write a review about the topic. AlfM, FV, and FB took care about the literature search. LD and NI wrote the manuscript with the supervision of MV, FS, and DA. FB and GC verified the accuracy of all pharmacological and molecular information given in the review. AC and AlbM contributed to the final manuscript and supervised the project. AC took care about the final form of the manuscript and made changes to adapt the review to journal guidelines. All authors contributed to the article and approved the submitted version.

## Conflict of Interest

The authors declare that the research was conducted in the absence of any commercial or financial relationships that could be construed as a potential conflict of interest.

## Publisher's Note

All claims expressed in this article are solely those of the authors and do not necessarily represent those of their affiliated organizations, or those of the publisher, the editors and the reviewers. Any product that may be evaluated in this article, or claim that may be made by its manufacturer, is not guaranteed or endorsed by the publisher.

## Abbreviations

DIP: drug-induced photosensitivity
UVR: ultraviolet radiation
UVA: ultraviolet A
UVB: ultraviolet B
NSAIDs: non-steroidal anti-inflammatorydrugs
DI-PTRs: drug-induced phototoxic reactions
DI-PARs: drug-induced photoallergic reactions
POMC: pro-opiomelanocortin
MSH: melanocyte stimulating hormone
PCT: porphyria cutanea tarda
CCB: calcium channel blockers
ROS: reactive oxygen species
NAD: nicotinamide adenine dinucleotide
PARP1: Poly (ADP-ribose) polymerase 1
EM: erythema multiforme
PEM: photodistributed erythema multiforme
PMLE: polymorphous light eruption
DILE: drug-induced lupus erythematosus
SCLE: subacute cutaneous subtype
DI-SCLE: drug-induced SCLE
DI-DLE: drug-induced discoid lupus erythematous
EGFR: epidermal growth factor receptor
MAPK: mitogen-activated protein kinases.

## References

[1] Moore DE. Drug-induced cutaneous photosensitivity: incidence, mechanism, prevention and management. Drug Saf. (2002) 25:345–72. 10.2165/00002018-200225050-00004 12020173

[2] Selvaag E. Clinical drug photosensitivity. a retrospective analysis of reports to the norwegian adverse drug reactions committee from the years 1970-1994. Photodermatol Photoimmunol Photomed. (1997) 13:21–3. 10.1111/j.1600-0781.1997.tb00103.x 9361124

[3] Hofmann GA Weber B. Drug-induced photosensitivity: culprit drugs, potential mechanisms and clinical consequences. J Dtsch Dermatol Ges. (2021) 19:19–29. 10.1111/ddg.14314 33491908 PMC7898394

[4] Kowalska J Rok J Rzepka Z Wrześniok D. Drug-Induced photosensitivity-from light and chemistry to biological reactions and clinical symptoms. Pharmaceuticals. (2021) 14:723. 10.3390/ph14080723 34451820 PMC8401619

[5] Duarte I Rotter A Malvestiti A Silva M. The role of glass as a barrier against the transmission of ultraviolet radiation: an experimental study. Photodermatol Photoimmunol Photomed. (2009) 25:181–4. 10.1111/j.1600-0781.2009.00434.x 19614895

[6] Blakely KM Drucker AM Rosen CF. Drug-induced photosensitivity-an update: culprit drugs, prevention and management. Drug Saf. (2019) 42:827–47. 10.1007/s40264-019-00806-5 30888626

[7] Elkeeb D Elkeeb L Maibach H. Photosensitivity: a current biological overview. Cutan Ocul Toxicol. (2012) 31:263–72. 10.3109/15569527.2012.656293 22338618

[8] Baptista MS Cadet J Di Mascio P Ghogare AA Greer A Hamblin MR. Type I and Type II photosensitized oxidation reactions: guidelines and mechanistic pathways. Photochem Photobiol. (2017) 93:912–9. 10.1111/php.12716 28084040 PMC5500392

[9] Epstein JH. Phototoxicity and photoallergy. Semin Cutan Med Surg. (1999) 18:274–84. 10.1016/S1085-5629(99)80026-1 10604793

[10] Allen JE. Drug-induced photosensitivity. Clin Pharm. (1993) 12:580–7. 8222522

[11] Tokura Y. Drug photoallergy. J CutanImmunol Allergy. (2018) 1:48–57. 10.1002/cia2.12017

[12] Inalöz HS Kirtak N Herken H Ozgöztaşi O Aynacioglu AS. Citalopram-induced photopigmentation. J Dermatol. (2001) 28:742–5. 10.1111/j.1346-8138.2001.tb00070.x 11804072

[13] Angel TA Stalkup JR Hsu S. Photodistributed blue-gray pigmentation of the skin associated with long-term imipramine use. Int J Dermatol. (2002) 41:327–9. 10.1046/j.1365-4362.2002.01479.x 12100685

[14] Eichenfield DZ Cohen PR. Amitriptyline-induced cutaneous hyperpigmentation: case report and review of psychotropic drug-associated mucocutaneous hyperpigmentation. Dermatol Online J. (2016) 22:13030/qt3455571b. 10.5070/D3222030090 27267189

[15] Calheiros T de Almeida HL Jr Jorge VM de Almeida AL Motta L. Light and electron microscopy of chlorpromazine-induced hyperpigmentation. J Cutan Pathol. (2020) 47:402–5. 10.1111/cup.13612 31714613

[16] Boyer M Katta R Markus R. Diltiazem-induced photodistributed hyperpigmentation. Dermatol Online J. (2003) 9:10. 10.5070/D33C97J4Z5 14996383

[17] Yones SS O'Donoghue NB Palmer RA Menagé Hdu P Hawk JL. Persistent severe amiodarone-induced photosensitivity. Clin Exp Dermatol. (2005) 30:500–2. 10.1111/j.1365-2230.2005.01820.x 16045677

[18] Ammoury A Michaud S Paul C Prost-Squarcioni C Alvarez F Lamant L. Photodistribution of blue-gray hyperpigmentation after amiodarone treatment: molecular characterization of amiodarone in the skin. Arch Dermatol. (2008) 144:92–6. 10.1001/archdermatol.2007.25 18209173

[19] Morissette G Ammoury A Rusu D Marguery MC Lodge R Poubelle PE. Intracellular sequestration of amiodarone: role of vacuolar ATPase and macroautophagic transition of the resulting vacuolar cytopathology. Br J Pharmacol. (2009) 157:1531–40. 10.1111/j.1476-5381.2009.00320.x 19594752 PMC2765325

[20] Kong HH Fine HA Stern JB Turner ML. Cutaneous pigmentation after photosensitivity induced by vandetanib therapy. Arch Dermatol. (2009) 145:923–5. 10.1001/archdermatol.2009.177 19687425 PMC3521518

[21] Epstein JH Tuffanelli DL Seibert JS Epstein WL. Porphyria-like cutaneous changes induced by tetracycline hydrochloride photosensitization. Arch Dermatol. (1976) 112:661–6. 10.1001/archderm.112.5.661 132139

[22] Bilsland D Douglas WS. Sunbed pseudoporphyria induced by nalidixic acid. Br J Dermatol. (1990) 123:547. 10.1111/j.1365-2133.1990.tb01464.x 2095188

[23] Kwong WT Hsu S. Pseudoporphyria associated with voriconazole. J Drugs Dermatol. (2007) 6:1042–4. 17966183

[24] Willis ZI Boyd AS Di Pentima MC. Phototoxicity, pseudoporphyria, and photo-onycholysis due to voriconazole in a pediatric patient with leukemia and invasive aspergillosis. J Pediatric Infect Dis Soc. (2015) 4:e22–4. 10.1093/jpids/piu065 26407422

[25] Breier F Feldmann R Pelzl M Gschnait F. Pseudoporphyria cutanea tarda induced by furosemide in a patient undergoing peritoneal dialysis. Dermatology. (1998) 197:271–3. 10.1159/000018012 9812036

[26] Motley RJ. Pseudoporphyria due to dyazide in a patient with vitiligo. BMJ. (1990) 300:1468. 10.1136/bmj.300.6737.1468-a 2379016 PMC1663168

[27] Parodi A Guarrera M Rebora A. Amiodarone-induced pseudoporphyria. Photodermatol. (1988) 5:146–7. 3050908

[28] McDonagh AJ Harrington CI. Pseudoporphyria complicating etretinate therapy. Clin Exp Dermatol. (1989) 14:437–8. 10.1111/j.1365-2230.1989.tb02606.x 2605807

[29] Riordan CA Anstey A Wojnarowska F. Isotretinoin-associated pseudoporphyria. Clin Exp Dermatol. (1993) 18:69–71. 10.1111/j.1365-2230.1993.tb00974.x 8440060

[30] Johnson OR Stewart MF Bakshi A Weston P. An unusual bullous eruption: olanzapine induced pseudoporphyria. BMJ Case Rep. (2019) 12:e232263. 10.1136/bcr-2019-232263 31678928 PMC6827790

[31] Taylor BJ Duffill MB. Pseudoporphyria from nonsteroidal antiinflammatory drugs. N Z Med J. (1987) 100:322–3. 3451093

[32] LaDuca JR Bouman PH Gaspari AA. Nonsteroidal antiinflammatory drug-induced pseudoporphyria: a case series. J Cutan Med Surg. (2002) 6:320–6. 10.1177/120347540200600402 12118363

[33] Nardi G Lhiaubet-Vallet V Miranda MA. Photosensitization by imatinib. a photochemical and photobiological study of the drug and its substructures. Chem Res Toxicol. (2014) 27:1990–5. 10.1021/tx500328q 25275675

[34] Goetze S Hiernickel C Elsner P. Phototoxicity of doxycycline: a systematic review on clinical manifestations, frequency, cofactors, and prevention. Skin Pharmacol Physiol. (2017) 30:76–80. 10.1159/000458761 28291967

[35] Elmas ÖF Akdeniz N. A case of doxycyclin-induced photo-onycholysis with dermoscopic features. Balkan Med J. (2020) 37:113. 10.4274/balkanmedj.galenos.2019.2019.11.22 31833717 PMC7094182

[36] Rutherford T Sinclair R. Photo-onycholysis due to indapamide. Australas J Dermatol. (2007) 48:35–6. 10.1111/j.1440-0960.2007.00324.x 17222300

[37] Bentabet Dorbani I Badri T Benmously R Fenniche S Mokhtar I. Griseofulvin-induced photo-onycholysis. Presse Med. (2012) 41:879–81. 10.1016/j.lpm.2011.11.014 22244720

[38] Mahajan VK Sharma NL. Photo-onycholysis due to sparfloxacin. Australas J Dermatol. (2005) 46:104–5. 10.1111/j.1440-0960.2005.00153.x 15842405

[39] Gregoriou S Karagiorga T Stratigos A Volonakis K Kontochristopoulos G Rigopoulos D. Photo-onycholysis caused by olanzapine and aripiprazole. J Clin Psychopharmacol. (2008) 28:219–20. 10.1097/JCP.0b013e318166c50a 18344734

[40] McCormack LS Elgart ML Turner ML. Benoxaprofen-induced photo-onycholysis. J Am Acad Dermatol. (1982) 7:678–80. 10.1016/S0190-9622(82)70151-3 7142476

[41] Al-Kathiri L Al-Asmaili A. Diclofenac-induced photo-onycholysis. Oman Med J. (2016) 31:65–8. 10.5001/omj.2016.12 26816569 PMC4720947

[42] Hanneken S Wessendorf U Neumann NJ. Photodynamic onycholysis: first report of photo-onycholysis after photodynamic therapy. Clin Exp Dermatol. (2008) 33:659–60. 10.1111/j.1365-2230.2008.02941.x 18801098

[43] Vaccaro M Calapai G Guarneri F Mannucci C Lentini M Cannavò SP. Photodistributed telangiectasia following use of escitalopram. Allergol Int. (2016) 65:336–7. 10.1016/j.alit.2016.01.004 26888670

[44] Borgia F Vaccaro M Guarneri F Cannavò SP. Photodistributed telangiectasia following use of cefotaxime. Br J Dermatol. (2000) 143:674–5. 10.1111/j.1365-2133.2000.03749.x 10971368

[45] Vaccaro M Borgia F Barbuzza O Guarneri B. Photodistributed eruptive telangiectasia: an uncommon adverse drug reaction to venlafaxine. Br J Dermatol. (2007) 157:822–4. 10.1111/j.1365-2133.2007.08082.x 17655739

[46] Vaccaro M Caradonna E Guarneri F Borgia F Cannavò SP. Photodistributed telangiectasia following the use of psychotropic drugs. Dermatol Ther. (2020) 33:e14237. 10.1111/dth.14237 32852114

[47] Bakkour W Haylett AK Gibbs NK Chalmers RJ Rhodes LE. Photodistributed telangiectasia induced by calcium channel blockers: case report and review of the literature. Photodermatol Photoimmunol Photomed. (2013) 29:272–5. 10.1111/phpp.12054 24001385

[48] Prabhu D Dawe RS Mponda K. Pellagra a review exploring causes and mechanisms, including isoniazid-induced pellagra. Photodermatol Photoimmunol Photomed. (2021) 37:99–104. 10.1111/phpp.12659 33471377

[49] Stevens HP Ostlere LS Begent RH Dooley JS Rustin MH. Pellagra secondary to 5-fluorouracil. Br J Dermatol. (1993) 128:578–80. 10.1111/j.1365-2133.1993.tb00240.x 8504053

[50] Thami GP Kaur S Kanwar AJ. Delayed reactivation of haloperidol induced photosensitive dermatitis by methotrexate. Postgrad Med J. (2002) 78:116–7. 10.1136/pmj.78.916.116 11807213 PMC1742246

[51] Oliveira A Sanches M Selores M. Azathioprine-induced pellagra. J Dermatol. (2011) 38:1035–7. 10.1111/j.1346-8138.2010.01189.x 21658113

[52] Milanesi N Gola M Francalanci S. Photosensitivity in drug induced pellagra. G Ital Dermatol Venereol. (2019) 154:366–7. 10.23736/S0392-0488.17.05776-5 29192472

[53] Gupta Y Shah I. Ethionamide-induced pellagra. J Trop Pediatr. (2015) 61:301–3. 10.1093/tropej/fmv021 25828829

[54] Johnston GA. Thiazide-induced lichenoid photosensitivity. Clin Exp Dermatol. (2002) 27:670–2. 10.1046/j.1365-2230.2002.01108.x 12472543

[55] Dawson TA. Quinine lichenoid photosensitivity. Clin Exp Dermatol. (1986) 11:670–1. 10.1111/j.1365-2230.1986.tb00531.x 3665157

[56] Wolf R Dorfman B Krakowski A. Quinidine-induced lichenoid and eczematous photodermatitis. Dermatologica. (1987) 174:285–9. 10.1159/000249200 2957249

[57] Jones HE Lewis CW Reisner JE. Photosensitive lichenoid eruption associated with demeclocycline. Arch Dermatol. (1972) 106:58–63. 10.1001/archderm.106.1.58 4261050

[58] Lee AY Jung SY. Two patients with isoniazid-induced photosensitive lichenoid eruptions confirmed by photopatch test. Photodermatol Photoimmunol Photomed. (1998) 14:77–8. 10.1111/j.1600-0781.1998.tb00017.x 9638730

[59] Hamanaka H Mizutani H Shimizu M. Sparfloxacin-induced photosensitivity and the occurrence of a lichenoid tissue reaction after prolonged exposure. J Am Acad Dermatol. (1998) 38:945–9. 10.1016/S0190-9622(98)70157-4 9632002

[60] Susong J Carrizales S. Lichenoid photosensitivity: an unusual reaction to doxycycline and an unusual response. Cutis. (2014) 93:E1–2. 24897144

[61] Horio T Yokoyama M. Tegaful photosensitivity–lichenoid and eczematous types. Photodermatol. (1986) 3:192–3. 3092199

[62] Shah RA Bennett DD Burkard ME. Photosensitive lichenoid skin reaction to capecitabine. BMC Cancer. (2017) 17:866. 10.1186/s12885-017-3882-4 29258457 PMC5735949

[63] Matsuo I Ozawa A Niizuma K Ohkido M. Lichenoid dermatitis due to chlorpromazine phototoxicity. Dermatologica. (1979) 159:46–9. 10.1159/000250560 383531

[64] Yasuda S Mizuno N Kawabe Y Sakakibara S. Photosensitive lichenoid reaction accompanied by nonphotosensitive subacute prurigo caused by carbamazepine. Photodermatol. (1988) 5:206–10. 3222169

[65] Llambrich A Lecha M. Photoinduced lichenoid reaction by thioridazine. Photodermatol Photoimmunol Photomed. (2004) 20:108–9. 10.1111/j.1600-0781.2004.00087.x 15030597

[66] Collazo MH Sánchez JL Figueroa LD. Defining lichenoid photodermatitis. Int J Dermatol. (2009) 48:239–42. 10.1111/j.1365-4632.2009.03887.x 19261010

[67] Kanwar AJ Dhar S Ghosh S. Photosensitive lichenoid eruption due to enalapril. Dermatology. (1993) 187:80. 10.1159/000247209 8324286

[68] Gardeazabal J Gonzalez M Izu R Gil N Aguirre A Diaz-Perez JL. Phenofibrate-induced lichenoid photodermatitis. Photodermatol Photoimmunol Photomed. (1993) 9:156–8. 8318433

[69] Dogra S Kanwar AJ. Clopidogrel bisulphate-induced photosensitive lichenoid eruption: first report. Br J Dermatol. (2003) 148:609–10. 10.1046/j.1365-2133.2003.05209_17.x 12653774

[70] Rodríguez-Pazos L Sánchez-Aguilar D Rodríguez-Granados MT Pereiro-Ferreirós MM Toribio J. Erythema multiforme photo induced by statins. PhotodermatolPhotoimmunol Photomed. (2010) 26:216–8. 10.1111/j.1600-0781.2010.00519.x 20626826

[71] Cohen PR. Photodistributed erythema multiforme: paclitaxel-related, photosensitive conditions in patients with cancer. J Drugs Dermatol. (2009) 8:61–4. 19180897

[72] Gutiérrez-González E Rodríguez-Pazos L Rodríguez-Granados MT Toribio J. Photosensitivity induced by naproxen. Photodermatol Photoimmunol Photomed. (2011) 27:338–40. 10.1111/j.1600-0781.2011.00625.x 22092742

[73] Izu K Hino R Isoda H Nakashima D Kabashima K Tokura Y. Photocontact dermatitis to ketoprofen presenting with erythema multiforme. Eur J Dermatol. (2008) 18:710–3. 10.1684/ejd.2008.0525 18955208

[74] Leroy D Le Maitre M Deschamps P. Photosensitive erythema multiforme apparently induced by phenylbutazone. Photodermatol. (1985) 2:176–7. 4022817

[75] Patri A Fabbrocini G Megna M Lauro W D'Onofrio P Gallo L. Itraconazole-induced photodistributed erythema multiforme. Dermatol Ther. (2021) 34:e14901. 10.1111/dth.14901 33605022

[76] Hamada K Sawada Y Yamaguchi T Ohmori S Omoto D Haruyama S. Photosensitivity due to tocilizumab presenting with erythema multiforme-like lesions. Eur J Dermatol. (2016) 26:503–4. 10.1684/ejd.2016.2819 27346710

[77] Zhang XM Nakagawa M Kawai K Kawai K. Erythema-multiforme-like eruption following photoallergic contact dermatitis from oxybenzone. Contact Dermatitis. (1998) 38:43–4. 10.1111/j.1600-0536.1998.tb05637.x 9504247

[78] Pretel M Marquès L España A. Drug-induced lupus erythematosus. Actas Dermosifiliogr. (2014) 105:18–30. 10.1016/j.adengl.2012.09.025 23164669

[79] Bataille P Chasset F Monfort JB De Risi-Pugliese T Soria A Francès C. Cutaneous drug-induced lupus erythematosus: clinical and immunological characteristics and update on new associated drugs. Ann Dermatol Venereol. (2021) 148:211–20. 10.1016/j.annder.2021.02.006 34711400

[80] Correia O Lomba Viana H Azevedo R Delgado L Polónia J. Possible phototoxicity with subsequent progression to discoid lupus following pantoprazole administration. Clin Exp Dermatol. (2001) 26:455–6. 10.1046/j.1365-2230.2001.00857.x 11488838

[81] Cohen PR. Discoid lupus erythematosus lesions associated with systemic fluorouracil agents: a case report and review. Cureus. (2020) 12:e7828. 10.7759/cureus.7828 32467803 PMC7249746

[82] Cemil BC Atas H Canpolat F Akca Y Sasmaz R. Infliximab-induced discoid lupus erythematosus. Lupus. (2013) 22:515–8. 10.1177/0961203313479423 23554040

[83] Brehon A Moguelet P Guégan S Abisror N Barbaud A Beal C. Discoid drug-induced lupus erythematosus induced by antitumor necrosis factor agents is a very rare subtype of cutaneous lupus: three cases and literature review. Dermatol Ther. (2020) 33:e13364. 10.1111/dth.13364 32239589

[84] Freedman JB Herskovitz I Maderal AD. Chronic cutaneous lupus erythematosus (discoid lupus) induced by palbociclib. Int J Dermatol. (2020) 59:e216–8. 10.1111/ijd.14716 31724158

[85] Krause W. Association of skin hyperpigmentation and drug use: a systematic review. G Ital Dermatol Venereol. (2016) 151:694–9. 27248149

[86] Khandpur S Porter RM Boulton SJ Anstey A. Drug-induced photosensitivity: new insights into pathomechanisms and clinical variation through basic and applied science. Br J Dermatol. (2017) 176:902–9. 10.1111/bjd.14935 27510322

[87] D'Mello SA Finlay GJ Baguley BC Askarian-Amiri ME. Signaling pathways in melanogenesis. Int J Mol Sci. (2016) 17:1144. 10.3390/ijms17071144 27428965 PMC4964517

[88] Hamblin MR Abrahamse H. Tetracyclines: light-activated antibiotics? Future Med Chem. (2019) 11:2427–45. 10.4155/fmc-2018-0513 31544504 PMC6785754

[89] Odorici G Monfrecola G Bettoli V. Tetracyclines and photosensitive skin reactions: A narrative review. Dermatol Ther. (2021) 34:e14978. 10.1111/dth.14978 33991382 PMC8459281

[90] Green JJ Manders SM. Pseudoporphyria. J Am Acad Dermatol. (2001) 44:100–8. 10.1067/mjd.2000.111338 11148469

[91] Dabski C Beutner EH. Studies of laminin and type IV collagen in blisters of porphyria cutanea tarda and drug-induced pseudoporphyria. J Am Acad Dermatol. (1991) 25:28–32. 10.1016/0190-9622(91)70169-3 1880250

[92] Maitra D Bragazzi Cunha J Elenbaas JS Bonkovsky HL Shavit JA Omary MB. Porphyrin-induced protein oxidation and aggregation as a mechanism of porphyria-associated cell injury. Cell Mol Gastroenterol Hepatol. (2019) 8:535–48. 10.1016/j.jcmgh.2019.06.006 31233899 PMC6820234

[93] Baran R Juhlin L. Photoonycholysis. Photodermatol Photoimmunol Photomed. (2002) 18:202–7. 10.1034/j.1600-0781.2002.00760.x 12390677

[94] Baran R Juhlin L. Drug-induced photo-onycholysis. three subtypes identified in a study of 15 cases. J Am Acad Dermatol. (1987) 17:1012–6. 10.1016/S0190-9622(87)70291-6 2963036

[95] Jerkic M Letarte M. Contribution of oxidative stress to endothelial dysfunction in hereditary hemorrhagic telangiectasia. Front Genet. (2015) 6:34. 10.3389/fgene.2015.00034 25763011 PMC4327735

[96] Gould JW Mercurio MG Elmets CA. Cutaneous photosensitivity diseases induced by exogenous agents. J Am Acad Dermatol. (1995) 33:551–73. 10.1016/0190-9622(95)91271-1 7673488

[97] Lamoreux MR Sternbach MR Hsu WT. Erythema multiforme. Am Fam Physician. (2006) 74:1883–8. 17168345

[98] Rodríguez-Pazos L Gómez-Bernal S Rodríguez-Granados MT Toribio J. Photodistributed erythema multiforme. Actas Dermosifiliogr. (2013) 104:645–53. 10.1016/j.adengl.2012.01.024 23962583

[99] Marzano AV Vezzoli P Crosti C. Drug-induced lupus: an update on its dermatologic aspects. Lupus. (2009) 18:935–40. 10.1177/0961203309106176 19762393

[100] Bruynzeel DP Ferguson J Andersen K Gonçalo M English J Goossens A. Photopatch testing: a consensus methodology for Europe. J Eur Acad Dermatol Venereol. (2004) 18:679–82. 10.1111/j.1468-3083.2004.01053.x 15482294

[101] Petersen B Wulf HC. Application of sunscreen–theory and reality. Photodermatol Photoimmunol Photomed. (2014) 30:96–101. 10.1111/phpp.12099 24313722

[102] Suozzi K Turban J Girardi M. Cutaneous photoprotection: a review of the current status and evolving strategies. Yale J Biol Med. (2020) 93:55–67. 32226337 PMC7087054

[103] Adam J. Sun-protective clothing. J Cutan Med Surg. (1998) 3:50–3. 10.1177/120347549800300115 9677262

[104] Gambichler T Laperre J Hoffmann K. The European standard for sun-protective clothing: EN 13758. J Eur Acad Dermatol Venereol. (2006) 20:125–30. 10.1111/j.1468-3083.2006.01401.x 16441617

[105] Tan KR Magill AJ Parise ME Arguin PM Centers for Disease Control and Prevention. Doxycycline for malaria chemoprophylaxis and treatment: report from the CDC expert meeting on malaria chemoprophylaxis. Am J Trop Med Hyg. (2011) 84:517–31. 10.4269/ajtmh.2011.10-0285 21460003 PMC3062442

[106] Moran C Zetler E. A review of smartphone applications for promoting sun protection practices. J Am Acad Dermatol. (2019) 81:613–5. 10.1016/j.jaad.2018.11.027 30471315

[107] Huang C Yan S Ren J Xiang L Hu Y Kang K. A quantitative assessment of the effects of formal sun protection education on photosensitive patients. Photodermatol Photoimmunol Photomed. (2013) 29:261–5. 10.1111/phpp.12065 24001382

[108] Steele C Burkhart C Tolleson-Rinehart S. “Live sun smart!” testing the effectiveness of a sun safety program for middle schoolers. Pediatr Dermatol. (2020) 37:504–9. 10.1111/pde.14141 32157728

[109] Walkosz BJ Buller D Buller M Wallis A Meenan R Cutter G. Sun safe workplaces: effect of an occupational skin cancer prevention program on employee sun safety practices. J Occup Environ Med. (2018) 60:900–7. 10.1097/JOM.0000000000001427 30095593 PMC6224296

[110] Xu Y Voorhees JJ Fisher GJ. Epidermal growth factor receptor is a critical mediator of ultraviolet B irradiation-induced signal transduction in immortalized human keratinocyte HaCaT cells. Am J Pathol. (2006) 169:823–30. 10.2353/ajpath.2006.050449 16936259 PMC1698809

[111] Vaccaro M Pollicino A Barbuzza O Guarneri B. Trichomegaly of the eyelashes following treatment with cetuximab. Clin Exp Dermatol. (2009) 34:402–3. 10.1111/j.1365-2230.2008.02842.x 19120397

[112] Zhang J Wang X Vikash V Ye Q Wu D Liu Y. ROS and ROS-mediated cellular signaling. Oxid Med Cell Longev. (2016) 2016:4350965. 10.1155/2016/4350965 26998193 PMC4779832

[113] Srinivas US Tan BWQ Vellayappan BA Jeyasekharan AD. ROS and the DNA damage response in cancer. Redox Biol. (2019) 25:101084. 10.1016/j.redox.2018.101084 30612957 PMC6859528

[114] Vaccaro M Cicero F Mannucci C Calapai G Spatari G Barbuzza O. IL-33 circulating serum levels are increased in patients with non-segmental generalized vitiligo. Arch Dermatol Res. (2016) 308:527–30. 10.1007/s00403-016-1675-2 27388717

[115] Vaccaro M Bagnato G Cristani M Borgia F Spatari G Tigano V. Oxidation products are increased in patients affected by non-segmental generalized vitiligo. Arch Dermatol Res. (2017) 309:485–90. 10.1007/s00403-017-1746-z 28551758

[116] Vaccaro M Irrera N Cutroneo G Rizzo G Vaccaro F Anastasi GP. Differential expression of nitric oxide synthase isoforms nnos and inos in patients with non-segmental generalized vitiligo. Int J Mol Sci. (2017) 18:2533. 10.3390/ijms18122533 29186858 PMC5751136

[117] Custurone P Di Bartolomeo L Irrera N Borgia F Altavilla D Bitto A. Role of cytokines in vitiligo: pathogenesis and possible targets for old and new treatments. Int J Mol Sci. (2021) 22:11429. 10.3390/ijms222111429 34768860 PMC8584117

